# Genital warts and infection with human immunodeficiency virus in high-risk women in Burkina Faso: a longitudinal study

**DOI:** 10.1186/1471-2334-11-20

**Published:** 2011-01-20

**Authors:** Andrea J Low, Tim Clayton, Issouf Konate, Nicolas Nagot, Abdoulaye Ouedraogo, Charlotte Huet, Marie-Noelle Didelot-Rousseau, Michel Segondy, Philippe Van de Perre, Philippe Mayaud

**Affiliations:** 1 London School of Hygiene & Tropical Medicine, London, UK; 2 Centre Muraz, Bobo-Dioulasso, Burkina Faso; 3 Université Montpellier 1, EA 4205 « Transmission, Pathogenèse et Prévention de l'Infection par le VIH »; and CHU Montpellier, Laboratoire de Bactériologie-Virologie and Département d'Information Médicale, Montpellier, France

## Abstract

**Background:**

Human papillomaviruses are the most common sexually transmitted infections, and genital warts, caused by HPV-6 and 11, entail considerable morbidity and cost. The natural history of genital warts in relation to HIV-1 infection has not been described in African women. We examined risk factors for genital warts in a cohort of high-risk women in Burkina Faso, in order to further describe their epidemiology.

**Methods:**

A prospective study of 765 high-risk women who were followed at 4-monthly intervals for 27 months in Burkina Faso. Logistic and Cox regression were used to identify factors associated with prevalent, incident and persistent genital warts, including HIV-1 serostatus, CD4+ count, and concurrent sexually transmitted infections. In a subset of 306 women, cervical HPV DNA was tested at enrolment.

**Results:**

Genital wart prevalence at baseline was 1.6% (8/492) among HIV-uninfected and 7.0% (19/273) among HIV-1 seropositive women. Forty women (5.2%) experienced at least one incident GW episode. Incidence was 1.1 per 100 person-years among HIV-uninfected women, 7.4 per 100 person-years among HIV-1 seropositive women with a nadir CD4+ count >200 cells/μL and 14.6 per 100 person-years among HIV-1 seropositive women with a nadir CD4+ count ≤200 cells/μL. Incident genital warts were also associated with concurrent bacterial vaginosis, and genital ulceration. Antiretroviral therapy was not protective against incident or persistent genital warts. Detection of HPV-6 DNA and abnormal cervical cytology were strongly associated with incident genital warts.

**Conclusions:**

Genital warts occur much more frequently among HIV-1 infected women in Africa, particularly among those with low CD4+ counts. Antiretroviral therapy did not reduce the incidence or persistence of genital warts in this population.

## Background

Human papillomaviruses (HPV) are the most common sexually transmitted infections (STI) worldwide, although they are often asymptomatic[1]. HPV infection is usually transient,[2] but persistence depends on HPV type and host immune status[3,4]. HPV types are divided into high-risk (HR-HPV)and low-risk (LR-HPV), depending on their potential for causing cervical and other ano-genital cancers[5]. The LR-HPV types HPV-6 and HPV-11 cause significant morbidity as the predominant causes of genital warts (GWs)[6,7]. Comparatively little is known about the epidemiology and natural history of LR-HPV infection and GWs in sub-Saharan Africa. LR-HPV prevalence shows considerable regional variation, such as 11% in the Gambia,[8] or 28% in Tanzania,[9] and is higher among individuals infected with the human immunodeficiency virus (HIV)[10].

HIV infection is known to alter the natural history of HPV infection. Patients co-infected with HIV-1 and HPV have a greater likelihood of HPV progression, with an increased risk for development of cervical neoplasia in immunosuppressed women[11]. HIV-1 infected patients with GWs can experience florid and prolonged clinical manifestations driven by an impaired CD4+ T-lymphocyte response and other forms of immune dysfunction[12,13]. Low CD4+ count (≤200 cells/μL) is the strongest independent predictor of infection with HR-HPV genotypes and is also associated with an increased risk of GWs[13,14]. The impact of highly active antiretroviral therapy (HAART) on GWs remains uncertain, with different statistical designs revealing different effects on incidence and persistence[3,15]. Other possibly contributing factors to GWs, such as other sexually transmitted or reproductive tract infections (STI/RTI), are poorly understood. Some studies suggest that STI/RTI increase the acquisition of HPV. However, these data are limited and predominantly from Western populations[13,15].

We have established a cohort of women at high risk for HIV and other STI/RTI in Burkina Faso, providing an opportunity to examine the epidemiology of various STI/RTI over a three-year period. The aims of this study were to determine the prevalence, incidence and persistence of GWs, and their association with HIV infection, immunosuppression, and other risk factors in an African setting.

## Methods

### Participants and study procedures

The Yérélon Cohort was established in 1998 to examine factors associated with HIV infection among professional female sex workers and other high-risk women in Burkina Faso and to design interventions to prevent infection. A new phase of enrolment began in December 2003 and continued until January 2005. Women were eligible for enrolment if they declared a history of at least one transactional sex act per week, were aged 16 years or older, and were willing to undergo HIV testing. Some women were recruited from local organizations for people living with HIV/AIDS, using the same criteria. All women provided written informed consent. The Yérélon Cohort research protocol was approved by the institutional review board at Centre Muraz, and the research ethics committees at the Burkina Faso Ministry of Health and the London School of Hygiene & Tropical Medicine.

Participants were followed approximately every 4 months until February 2006 for a maximum of 6 follow-up visits. At enrolment, an interviewer administered a questionnaire eliciting socio-demographic and behavioral characteristics, and sexual health information. At subsequent visits, intervening behavioral, sexual health and treatment information was collected. At all visits a study clinician performed a full physical exam and collected genital samples. Clinicians documented the presence of abnormal discharge, genital ulcers, and GWs, which were defined as a genital proliferation with the clinical appearance of condylomata acuminata[15]. The genital sites were described as the cervix, vagina, labia, or 'external' (outer pubic and anal) areas. Patients were treated for STI/RTI according to national guidelines; there was no treatment available for GWs. Since April 2004, HIV-infected participants meeting the World Health Organization (WHO) eligibility criteria for HAART initiation in developing countries have been provided with antiretrovirals[16]. Women with HIV clinical stages III or IV or with a CD4+ count ≤200 cells/μL were treated with standard antiretroviral therapy, primarily zidovudine (AZT), lamivudine (3TC) and efavirenz (EFV). Women already on HAART at the enrolment visit had been provided with medications at the Bobo-Dioulasso University Hospital.

### Laboratory investigations

Vaginal smears were examined using wet-mount microscopy to detect *Trichomonas vaginalis *(TV) and Gram-stained to diagnose bacterial vaginosis (BV)[17]. Diagnosis of *Neisseria gonorrhoeae *was made by culture of cervical swabs using modified Thayer-Martin media. *Chlamydia trachomatis *diagnosis could not be performed during the study, but has been shown to be rare in this population[18].

Blood samples were collected at enrolment and then every 4 months. Syphilis serostatus was determined every 12 months using the rapid plasma reagin test (RPR, Human GmbH) confirmed with a *Treponema pallidum *haemagglutination assay (TPHA, Newmarket Laboratories). HSV-2 serology was done using the Kalon IgG2-ELISA test (Kalon Biologicals). HIV serology was done at enrolment and every 4 months among seronegative women using a Determine-1/2 rapid testing kit (Abbott Laboratories) with Genie-II (Bio-Rad Laboratories) confirmation. CD4+ counts were determined using FACScan (Becton Dickinson) at enrolment and every 6 months among HIV-1 seropositive women; plasma HIV-1 RNA was quantified every 6 to 12 months using real-time PCR with a lower limit of detection of 300 (2.48 log_10 _) copies/mL[19]. Values were converted to log_10 _copies/mL; those below the threshold of detection were assigned a value of half the threshold.

A subset of women enrolled between December 2003 and March 2004 submitted an additional cervical swab at enrolment for HPV DNA detection and liquid-based cytology using a Cervex swab and the ThinPrep 2000 processor (Cytyc Corporation)[10]. HPV genotyping was performed using the INNO-LiPA genotyping v2 test (Innogenetics)[20]. The 2001 Bethesda classification was used for Papanicolaou-stained cervical slides interpretation[21].

### Definitions

Prevalent GWs were defined as the presence of clinically-defined condylomata acuminata at the enrolment visit. Due to the small number of women with CD4+ counts at the enrolment visit, women were classified as HIV seronegative and HIV-1 seropositive at baseline. Incident GWs were defined as the first documented occurrence of condylomata acuminata in a previously undocumented site after the enrolment visit. Women with a GW in one location who developed a lesion in a new location were included as a new incident event. Persistent GWs were defined as GWs present in the same anatomical site for at least two consecutive visits, which could include the enrolment visit. Women were stratified during study follow-up according to their nadir CD4+ count in the following categories: (i) HIV-negative, (ii) HIV-1 positive with a nadir CD4+ count >200 cells/μL, and (iii) HIV-1 positive with a nadir CD4+ count ≤200 cells/μL.

### Statistical analysis

Analyses examined the association between HIV status and other potential risk factors with prevalent GWs. Univariate associations were examined using chi-squared tests for categorical variables and two-sample t-tests for continuous variables. Due to the strong associations between many of our variables of interest, a multivariate logistic regression model was developed in a stepwise fashion including HIV status and risk factors either decided a priori or independently associated with prevalent GWs using p < 0.05 as the inclusion criterion.

We measured incidence and persistence of GWs and determined associations of potential risk factors with these outcomes using Cox regression. To allow for intra-subject correlation, these models used shared frailty[22]. Other potential risk factors included time-updated measures, such as concurrent STI/RTI, use of HAART, and behavioral measures recorded at follow-up visits. In order to establish the most likely ordering of events, measures of behavior and confirmed STI/RTI over the preceding period were taken from the visit at which incident or persistent GWs were identified and serological time-updated measures were taken from the visit preceding any visit at which a new GW was identified. For HIV-1 seropositive women, a separate model was constructed which included CD4+ count and HAART use at the prior visit and maximum viral load during the study period. Nadir CD4+ count was not included in these models because model convergence could not be achieved.

Further analyses were conducted to examine the association of cervical HPV and squamous intraepithelial lesions (SIL) with incident GWs among women from whom cervical HPV DNA had been collected, using the same methods as above to construct the final model.

The effect of time in the study was assessed for each variable in all models and an interaction parameter was included if the hazard ratio changed significantly after one year of follow-up.

Statistical analyses were performed using Stata version10.0 (StataCorp).

## Results

### Characteristics of cohort participants

Overall, 767 women were enrolled in the Yérélon Cohort study between December 2003 and January 2005, and followed up until February 2006. Two hundred and sixty eight (34.9%) women were infected with HIV-1 and five (0.7%) were co-infected with HIV-1 and HIV-2. Two women were infected with HIV-2 alone, and were excluded from analyses. Characteristics of study participants at enrolment are presented in Table 1. Overall mean age was 28 years (range, 15-54). The median follow-up time was 1.7 years (range 0-2.2 years). At baseline, a quarter (24.7%, 66/267) of HIV-1 seropositive women had a CD4+ count <200 cells/μL and 9.5% (26/273) of women were on HAART. HIV-1 infected women were significantly older, were more likely to have been married, had fewer recent sexual partners and were less likely to use hormonal contraception. No women were infected with *Neisseria gonorrhoeae*.

**Table 1:** Characteristics of 765 high-risk women at cohort enrolment, according to HIV-1 serostatus

Characteristic	HIV seronegative (N = 492) n (%)	HIV-1 seropositive (N = 273) n (%)	**P-value**^**a**^
Age groups, years (N = 759)			
16-24	265 (54.1)	53 (19.7)	<0.001^b^
25-34	138 (28.2)	124 (46.1)	
≥35	87 (17.8)	92 (34.2)	
Education (N = 752)			
None	189 (38.8)	116 (44.8)	0.19
Primary or above	298 (61.2)	149 (56.2)	
Marital status (N = 754)			
Widowed	14 (2.9)	55 (20.8)	<0.001^a^
Divorced/Separated	70 (14.3)	50 (18.9)	
Married/cohabitating	88 (18.0)	58 (21.9)	
Single	317 (64.8)	102 (38.5)	
Age at first sex, median (IQR), years	16 (15-18)	16 (15-18)	0.42^b^
Number of sex partners in last week (N = 752)			
None	110 (22.5)	114 (43.2)	<0.001^b^
1-9	309 (63.3)	120 (45.5)	
≥10	69 (14.1)	30 (11.4)	
Currently smoking (N = 759)	25 (5.1)	19 (7.0)	0.30
Contraceptive use (N = 760)			
Other/None	405 (83.2)	249 (91.2)	0.002
Hormonal contraception	68 (14.0)	19 (7.0)	
Injectable contraception	14 (2.8)	5 (1.8)	
Practices regular vaginal douching	424 (86.2)	222 (81.3)	0.24
Condom use with clients/partners (N = 751)			
Sometimes/Never	177 (36.0)	98 (35.9)	
Always	315 (64.0)	175 (64.1)	0.98
Current genital warts	8 (1.6)	19 (7.0)	<0.001
Bacterial vaginosis (N = 718)	178 (37.2)	118 (49.2)	0.003
*Trichomonas vaginalis *(N = 596)	29 (7.2)	19 (9.6)	0.32
HSV-2 positive serology (N = 689)	226 (49.6)	204 (88.3)	<0.001
Current antiretroviral therapy (HAART)		26 (9.5)	
CD4+ count, cells/μL :			
Not on HAART (N = 242)			
>500		81 (33.5)	0.09^a,e^
200-500		105 (43.4)	
<200		56 (23.1)	
On HAART (N = 25)			
>500		4 (16.0)	
200-500		11 (44.0)	
<200		10 (40.0)	
Plasma HIV-1 RNA, log_10 _copies/mL ^d^:			
Not on HAART (N = 233)			
≥2.48		204 (87.6)	<0.001^a,e^
<2.48		29 (12.4)	
On HAART (N = 24)			
≥2.48		7 (29.2)	
<2.48		17 (70.82)	
Cervical HPV-6 or -11 (N = 306)^f^	13 (7.1)	15 (12.2)	0.13
LSIL (N = 306)^f^	17 (9.4)	48 (41.4)	<0.001
HSIL (N = 306)^f^	1 (0.6)	11 (9.5)	

IQR, interquartile range; HAART, highly active antiretroviral therapy; CI, confidence interval; HPV, human papillomavirus; LSIL/HSIL, low/high grade squamous intraepithelial lesions.

^a ^Determined by χ^2 ^analysis; ^b ^Determined by χ^2 ^test of trend; ^c ^Nonparametric equality of medians test; ^d ^Lower limit of detection of HIV-1 PVL is 2.48 log_10 _copies/mL; ^e ^Comparison of women on HAART vs not on HAART; ^f ^Conducted in subset of women.

In the analysis of the 306 women from whom cervical cytological samples were collected, HIV-1 seropositive women had significantly more cervical squamous intraepithelial lesions than HIV-uninfected women (P < 0.001) (Table 1). There were no cases of atypical squamous cells-cannot exclude HSIL (ASC-H), atypical glandular cells (AGC), or cancer identified.

### Prevalent genital warts and associated risk factors

At enrolment, 27 women (3.5%) had GWs, 1.6% (8/492) among HIV-uninfected and 7.0% (19/273) among HIV-1 seropositive women (P < 0.001) (Table 1). There were no prevalent GWs among the 26 women taking HAART. In the multivariate analysis, which included HSV-2 serostatus, the presence of bacterial vaginosis, number of recent sexual partners and age group, there was a 5-fold increase in risk for prevalent GWs in HIV-1 seropositive women (adjusted odds ratio [aOR] 5.33, 95%CI: 1.97 to 14.40, P = 0.001). Women who smoked had a 3-fold increased risk of prevalent GW (aOR 3.55, 95%CI: 1.03 to 12.32, P = 0.05). For HIV-1 seropositive women, there was no evidence of a relationship between CD4+ count and prevalence of GWs (CD4+ count >200 cells/μL vs. ≤200 cells/μL, [aOR = 1.77, 95%CI: 0.36 to 8.61, P = 0.48). None of the other variables shown in Table 1 were associated with prevalent GWs.

### Incident genital warts and associated risk factors

Over the 27 months of the study, 16.9% (129/765) of women were lost to follow-up. Twenty-one women died, all of whom were HIV-1 seropositive. The percentage of scheduled visits attended was 83.3%; this did not differ by HIV-1 serostatus.

Forty women (5.2%) experienced at least one incident GW episode for a total of 50 episodes. The most common sites for new lesions were the external genitalia (including the perianal area) observed in 28 women. GW incidence was 1.1 per 100 person-years among HIV-uninfected women, 7.4 per 100 person-years among HIV-1 seropositive women with a nadir CD4+ count >200 cells/μL and 14.6 per 100 person-years among HIV-1 seropositive women with a nadir CD4+ count ≤200 cells/μL (Table 2). Incidence was 16.7 per 100 person-years for women on HAART.

**Table 2:** Univariate and multivariate assessment of factors associated with incident genital warts over three years

Characteristic	Number of events/person-years	**Unadjusted HR**^**a **^**(95% CI)**	**Adjusted HR**^**b **^**(95% CI)**	P-value
**1. Among all women:**				
HIV status, nadir CD4+ count				
HIV seronegative	8/756	1.0	1.0	
HIV-1+, CD4 >200cells/μL	18/245	7.29 (3.0-17.70)	6.51 (2.38-17.78)	<0.001
HIV-1+, CD4 ≤200 cells/μL	24/165	14.92 (6.21-35.85)	19.13 (6.94-52.71)	<0.001
**Enrolment variables**:				
Age groups, years				
16-24	9/454	1.0	1.0	
25-34	28/414	3.29 (1.40-7.74)	1.74 (0.69-4.42)	0.24
>34	13/294	2.19 (0.84-5.72)	0.83 (0.29-2.37)	0.73
Education				
None	32/510	1.0		
Primary/Above	18/655	0.43 (0.22-0.86)	0.45 (0.23-0.88)	0.02
Age at first sex, years				
10-17	41/889	1.0		
18-29	9/275	0.71 (0.30-1.67)		
Smoking	5/63	1.76 (0.50-6.15)	1.56 (0.50-4.86)	0.44
HSV-2 positive serology	43/676	4.33 (1.81-10.38)	0.86 (0.31-2.38)	0.77
History of pregnancy	47/954	3.42 (0.96-12.16)	1.30 (0.33-5.11)	0.71
**Concurrent variables**:				
Number of sex partners in past week				
0	10/241	1.0	1.0	
1-9	30/753	1.05 (0.48-2.27)	1.46 (0.66-3.22)	0.35
≥10	10/116	2.44 (0.85-7.05)	3.16 (1.11-8.95)	0.03
Contraceptive use				
None/Other	40/958	1.0	1.0	
Hormonal contraception	4/156	0.64 (0.21-1.94)	0.85 (0.28-2.61)	0.77
Injectable contraception	4/34	2.95 (0.81-10.75)	3.24 (0.91-11.51)	0.07
Practices regular vaginal douching	43/987	1.01 (0.33-3.11)		
Condom use with clients/partners				
Sometimes/Never	31/799	1.0		
Always	19/311	1.75 (0.92-3.33)		
Days since last menstruation^c^				
≤15	27/503	1.0		
>15	14/415	0.69 (0.35-1.37)		
Bacterial vaginosis	18/226	2.11 (1.12-4.00)	2.17 (1.13-4.18)	0.02
*Trichomonas vaginalis*	1/30	1.18 (0.13-10.53)		
Genital ulceration	5/23	4.66 (1.55-14.03)	3.29 (1.13-9.55)	0.03
**2. Among HIV-1 positive women only**:				
On HAART at prior visit	15/94	1.39 (0.66-2.95)	1.50 (0.61-3.70)	0.38
Highest log_10 _viral load in the past 3 years		1.35 (0.99-1.83)	1.13 (0.78-1.65)	0.51
CD4+ count at prior visit (per 100 cells/μL increase)		0.73 (0.57-0.93)	0.79 (0.61-1.02)	0.07

HR, hazard ratio; CI, confidence interval; HAART: highly active antiretroviral therapy.

^a ^Determined by Cox regression with shared frailty; ^b ^Adjusted for HIV-1 serostatus and nadir CD4+ count, age group, educational status, smoking, history of pregnancy and HSV-2 serostatus at enrolment, and concurrent number of weekly sex partners, contraception, genital ulcerations and bacterial vaginosis; ^c ^Women with amenorrhea and menopause were excluded from this group.

In the multivariate model including all women, HIV-1 and nadir CD4+ count were the strongest predictors of incident GWs. Compared to HIV-uninfected women, HIV-1-seropositive women with a nadir CD4+ ≤200 cells/μL had an almost 20-fold higher risk of incident GWs (adjusted hazard ratio [aHR] 19.13, 95%CI: 6.94 to 52.71, P < 0.001); among HIV-1-seropositive women with CD4+ counts >200 cells/μL, the estimated risk was 6-fold higher (aHR 6.51, 95%CI: 2.38 to 17.78, P < 0.001). Having a primary education or above was protective (aHR 0.45, 95%CI: 0.23 to 0.88, P = 0.02). For the time-updated variables, concurrent bacterial vaginosis (aHR 2.17, 95%CI: 1.13 to 4.18, P = 0.02), and genital ulceration (aHR 3.29, 95%CI: 1.13 to 9.55, P = 0.03) were associated with incident GWs.

The model among HIV-1 seropositive women indicated a trend towards a protective effect for increasing CD4+ count at the prior visit (aHR 0.79 for each 100 cells/μL increase, 95%CI: 0.61 to 1.02, P = 0.07), but the use of HAART *per se *was not protective. High maximum HIV-1 plasma viral load was predictive of incident GWs, although this was not statistically significant. There was no evidence of an interaction between nadir CD4+ count and time since enrolment on incidence of GWs.

### Persistence of genital warts

In the absence of treatment, 40% of women with an identified GW (16/40) had persistent warts at the next visit. The strongest predictor of GW persistence was HIV-1 status and nadir CD4+ count: GW persistence was 0.4 per 100 person-years among HIV-uninfected women, 6.1 per 100 person-years among HIV-1 seropositive women with nadir CD4+ counts >200 cells/μL, and 13.4 per 100 person-years for those women with nadir CD4+ counts ≤200 cells/μL. The multivariate model among HIV-1 seropositive women indicated that there was no protective effect of prior antiretroviral treatment on persistence (aHR 1.71, 95%CI: 0.40 to 7.23, P = 0.47), but there was weak evidence that a CD4+ count was protective (aHR 0.72 for each 100 cells/μL increase, 95%CI: 0.45 to 1.14, P = 0.16). No other factors were significantly associated with persistence of GWs.

### Association of HPV-6 or -11 with incident genital warts

In the subgroup of 306 women with enrolment cytological and HPV DNA samples, HPV-52, a HR-HPV, was the most common HPV type, identified in 14.7% (45/306) of women. LR-HPV prevalence was 32.7% (100/306); the prevalence of HPV-6 and -11 were 6% (18/306) and 4% (13/306), respectively, and 71% (20/28) of women with HPV-6 or -11 were infected with more than one type of HPV. Detection of cervical HPV-6 DNA was associated with prevalent GWs (aOR 4.12, 95%CI: 1.17 to 14.53, P = 0.03) and had a strong predictive effect for incident GWs (aHR 9.09, 95% CI: 1.75 to 47.29, P = 0.009) during the first year of follow-up, compared to those without HPV-6 (Table 3). In fact, 44.4% (8/18) of the women who were positive for cervical HPV-6 DNA had either a prevalent or incident GWs during the study period. In the analysis of individual HPV types, there was weak evidence of an association between incident GWs and cervical HPV-52 DNA at enrolment (aHR 3.81, 95% CI: 0.90 to 16.23, P = 0.07), with one lesion occurring in a woman co-infected with HPV-6. There was no evidence for any association between GWs and other HPV types, including HPV-11, although numbers were very small. The presence of abnormal cervical cytology (LSIL or higher) at enrolment was also predictive of incident GWs (aHR 3.05, 95% CI: 1.17 to 7.92, P = 0.02); these relationships did not vary with time. None of the women negative for HPV DNA at baseline had an incident event. The relationship between HIV-1 status and incident GWs persisted after adjusting for the presence of HPV-6 or -11 at baseline. HAART did not protect against incident lesions (aHR 1.03, 95%CI: 0.26 to 4.05, P = 0.96).

**Table 3:** Associations between cervical HPV DNA, HIV-1 serostatus and cytology and incident genital warts among a sub-group of 306 women

Characteristic	Number of events/person-years	**Unadjusted HR**^**a **^**(95% CI)**	**Adjusted HR**^**b **^**(95% CI)**	P-value
Cervical HPV DNA at baseline				
HPV-6 positive, yes vs. no				
Effect in 1^st ^year of follow-up	4/17	8.67 (1.86-40.40)	9.09 (1.75-47.29)	0.009
Effect after 1^st ^year of follow-up	1/12	0.12 (0.03-0.54)	0.11 (0.02-0.57)	0.009
HPV-11 positive, yes vs. no	1/21	0.74 (0.07-7.58)	1.17 (0.11-12.37)	0.89
HPV-52 positive, yes vs. no	9/50	3.73 (1.28-10.83)	3.81 (0.90-16.23)	0.07
Other HPV types, negative for types 6, 11,52	19/226	1.48 (0.64-3.45)	2.64 (0.62-11.33)	0.19
Cervical cytology at baseline (N = 296)				
Normal	9/369	1.0	1.0	
ASCUS/LSIL/HSIL	23/134	7.38 (3.10-17.56)	3.05 (1.17-7.92)	0.02
HIV status, nadir CD4+ count				
HIV seronegative	4/289		1.0	
HIV-1+, CD4 >200cells/μL	11/119		3.22 (0.71-14.59)	0.13
HIV-1+, CD4 ≤200 cells/μL	18/79		10.94 (2.60-46.12)	0.001

Note. HR: hazard ratio; CI, confidence interval; ASCUS, atypical squamous changes of unknown significance; LSIL/HSIL, low/high squamous intraepithelial lesions.

^a ^Determined by Cox regression with shared frailty; ^b ^Model also included age group, history of pregnancy, history of smoking, hormonal contraception, education less than or greater than primary and number of sexual partners in past week. For those factors not conforming to proportional hazards, a parameter was added to determine the impact in the first year of follow-up compared to after one year.

## Discussion

This study provides the first longitudinal data on the natural history of GWs among HIV infected women in Africa. In this setting, we found an overall prevalence of GWs of 3.5%. This is consistent with GW prevalence from other countries in Africa,[24] although this depends on the region, prevalence of HIV-1, and the sexual risk behaviours of studied populations. The Women's Interagency HIV Study in the USA reported GW incidences of 2.2 per 100 person-years in HIV-uninfected and 8.9 per 100 person-years in HIV-1 seropositive women, which are similar to our findings [13].

As in studies in developed countries, HIV-1 infection and immune function, as measured by nadir CD4+ count, were the most powerful predictors of incident and persistent GWs,[13,15] even after adjusting for the presence of HPV. Although only partially understood, GW regression is thought to be dependent upon the cell-mediated immune response; CD4+ T-lymphocytes have been shown to be present in large numbers in the stroma under the lesions and in the regressing warts themselves, but their role in clearance has not been entirely elucidated[12,25]. In studies of canine oral papillomavirus, it is the response of CD4+ cells to viral proteins E6 and E7 that is responsible for regression of lesions[26]. The strong association between concurrent BV and genital ulceration and incident GWs supports a common determinant. There is good evidence supporting the role of the vaginal flora in both susceptibility to and clearance of infections, as HPV infects via micro-abrasions in the genital mucosa[27].

This is the first study to evaluate the impact of HAART on GWs in women in Africa. We did not detect any protective effect of HAART on incidence or persistence, despite the overall excellent adherence achieved in this cohort[28]. The burden of HR-HPV was recently shown to be reduced by effective HAART,[29] but the impact on LR-HPV is unknown, and its effect on GWs varies between studies[3,15]. However, our interpretation of these results is limited by the relatively small number of visits for women on HAART, and the partial data on concurrent CD4+ count and HIV-1 viral loads.

We identified a strong association between detection of cervical HPV-6 DNA and incident GWs in the first year of follow-up. Our data also support studies demonstrating a relative lack of epidemiological contribution of HPV-11 in GWs[7,30]. HPV-52 has been identified as one of the types most commonly identified in GWs in conjunction with HPV-6 in Australia[7]. Furthermore, a study of HPV in GWs in France demonstrated that 6.4% of patients with GWs had HPV-52 on cytobrush sampling[6]. It is probable that cervical HPV was only partially representative of causative genotypes for GWs; the trend towards an increase in GWs in women infected with other types of HPV could reflect local but undetected infection with HPV-6 or 11. Furthermore, the small number of women infected with HPV-11 limits the power of this study to detect an association. However, as there are few studies examining the distribution of HPV in GWs in Africa, it is possible that this reflects a different epidemiology of HPV in this region compared to other settings. Further studies with larger numbers of women and direct sampling of warts for HPV types are required to better understand this relationship.

Study limitations include the varying time between visits, and the broad categorization of anatomical location which might have led to undetected new GWs and thus an underestimate of incidence. Although rare in this setting, it is also possible that some women sought treatment for GWs elsewhere, which may have resulted in underestimates of GW persistence.

## Conclusions

This is the first study to examine the natural history of genital warts in African women and the impact of HIV-1 infection and HAART. This high-risk population with high HIV prevalence and GW incidence and little access to ablative therapy may contribute more to HPV transmission and suffer disproportionately from the consequences of HPV infection. We have demonstrated a high incidence of genital warts in this population, and a very strong association with immune suppression from HIV-1 infection; there was no protective effect from HAART. This and other epidemiological studies of HPV types in different populations should inform further vaccine development designed for African women.

## Competing interests

The authors declare that they have no competing interests.

## Authors' contributions

All authors have read and approved the final manuscript. AL contributed to the study design, data/statistical analyses and the drafting of the manuscript. TC contributed to the statistical analyses and interpretation of results, and the drafting of the manuscript. IK contributed to the study design and data collection, and drafting of the manuscript. NN contributed to the study design and data collection, and the drafting of the manuscript. AO contributed to the study design, data management and the drafting of the manuscript. CH contributed to the study design, data analyses and the drafting of the manuscript. MD-R performed the collection and processing of the HPV samples and contributed to the drafting of the manuscript. MS contributed to the study design, the data analyses and the interpretation of results, and the drafting of the manuscript. PVdP contributed to the study design, the interpretation of results, and the drafting of the manuscript. PM was the lead investigator of this project, and contributed to the analyses, interpretation of results, and drafting of the manuscript.

## Pre-publication history

The pre-publication history for this paper can be accessed here:

http://www.biomedcentral.com/1471-2334/11/20/prepub
